# Editorial on Special Issue “Precision Delivery of Drugs and Imaging Agents with Peptides”

**DOI:** 10.3390/pharmaceutics14030486

**Published:** 2022-02-23

**Authors:** Kaido Kurrikoff, Tambet Teesalu

**Affiliations:** 1 Institute of Technology, University of Tartu, Nooruse 1, 50411 Tartu, Estonia; 2 Laboratory of Precision and Nanomedicine, Department of Biomedicine and Translational Medicine, University of Tartu, Ravila 14b, 50411 Tartu, Estonia; 3 Center for Nanomedicine, Department of Cell, Molecular and Developmental Biology, University of California at Santa Barbara, Santa Barbara, CA 93106, USA

Homing peptides and cell-penetrating peptides allow for systemic targeting of diseased tissues and/or efficient intracellular delivery of payloads. The translational potential of peptides as targeting ligands is strengthened by their small size, low immunogenicity, and biocompatibility. Whereas early studies on homing and cell-penetrating peptides focused on cancer delivery, a number of other disease indications have been demonstrated to be targetable with the peptides for improved diagnosis and therapy.

The original contributions in this Special Issue address the development of internalizing peptides as facilitators of intracellular cargo uptake [1,2,3] as well as preclinical studies on homing peptide-guided molecular and nanoscale precision systemic drug delivery systems [4,5,6,7,8]. This thematic issue illustrates the current trends in the field of peptide-mediated affinity targeting. The first is expansion of the range of target pathologies beyond cancer, illustrated by studies using CAR peptide as an affinity ligand to target retinal diseases [4] and cystic fibrosis lesions [5]. The second trend is increased reliance on the disease-associated isoforms of the extracellular matrix as targets for homing peptides [3,4,5,6]. Extracellular matrix has been emerging, in many respects, as an ideal target for affinity delivery due to its robust upregulation in reactive tissues, abundance, low shedding profile, and accessibility to circulating probes.

The field of peptide-guided drug delivery is a vibrant multidisciplinary research area and this Special Issue serves to present a number of important advances in as outlined below.

An intriguing study from the laboratory of Dr. Hongbo Pang at University of Minnesota (USA) investigated whether a co-incubation with the cell-penetrating peptide transportan can facilitate cellular uptake of cargo compounds [1]. This mode of “bystander” delivery (in the past applied in the case of C-end Rule peptides such as clinical-stage iRGD peptide) is particularly appealing as it allows improved delivery of non-modified approved cancer drugs and imaging agents. Here, the authors demonstrated using in vitro and ex vivo cellular uptake models that co-incubate with transportan facilitates uptake of several types of nanoparticles. These findings improve the understanding of transportan-assisted cell entry, and suggest a simple mode to apply this peptide for intracellular delivery on approved unmodified nanoscale drugs.

A study by Dr. Rafael Morán-Torres et al. at National Autonomous University of Mexico deals with development of short selective cell-penetrating peptides, referred to as “moonlighting peptides” [2]. The authors apply machine-learning-based approach that they have reported previously to design new short selective cell-penetrating peptides and provide experimental evidence that resulting “moonlighting peptides” penetrate selectively in target mammalian and yeast cells.

In another original report in the current Special Issue [3], the team of Dr. Tambet Teesalu at University of Tartu (Estonia) reports mapping of the binding sites of the PL1, a bispecific tumor extracellular matrix (ECM) binding peptide, on its receptor molecules (Fibronectin EDB and Tenascin-C C-domains). This mechanistic understanding can be used for further improvement of the PL1 based targeting paradigm. Furthermore, the study revealed that the PL1 peptide-functionalized nanoparticles are taken up by cultured cancer cells. This feature expands the range of PL1 therapeutic payloads to intracellularly acting anticancer agents and radionuclides, including different anticancer drugs.

Two studies in this Special Issue deal with applications of homing peptide called CAR (amino acid sequence: CARSKNKDC), which was originally reported to target neovasculature in injured regenerating tissues. CAR recognizes the unique sulfation pattern of heparan sulfate proteoglycans on the target cells. The first study [4] coordinated by Dr. Tero Järvinen at Tampere University (Finland) addresses the relevance of the CAR peptide for targeting retinal diseases—a major cause for blindness in Western countries. The pharmacological treatment of these devastating diseases is hindered by poor bioavailability of drugs to the retina. The authors show that upon systemic administration, CAR peptide is targeted to the aberrant angiogenesis lesions of the oxygen-induced retinopathy, the experimental model of human retinopathies. Furthermore, the authors demonstrate that in oxygen-induced retinopathy mice, systemic CAR peptide is also home to lesions of bronchopulmonary dysplasia.

In the second study on CAR-mediated systemic targeting, Iqbal et al. used the peptide for precision delivery of therapeutic antifibrotic TGF-β modulator Decorin to skeletal muscle to slow progression of a mouse model of muscular dystrophy [5].

Development of contrast agents remains challenging due to lack of sensitivity, low signal to noise ratio, and/or difficulty in tissue penetration in the case of optical imaging agents. The work by the team of Dr. Juliana Hamzah and colleagues from the Harry Perkins Institute of Medical Research and Curtin University (Australia) reported development of iron oxide nanoparticles functionalized with CSG peptide to detect tumor stroma, based on the over-expression of laminin-nidogen-1 complex that is recognized specifically by the CSG peptide [6]. The authors showed that using this strategy, solid tumors, irrespective of their size and extent of vascularization, can be visualized by magnetic resonance imaging for their content of desmoplastic stroma.

In a paper by Hingorani et al., the authors report development of activatable cell-penetrating peptide conjugate for delivery of resiquimod, a TLR 7/8 agonist that has antitumor efficacy [7]. The authors demonstrate that upon systemic administration in syngeneic B16F10 melanoma mice, the cell-penetrating peptide module is proteolytically activated in malignant tissue. For activatable cell-penetrating peptide-resiquimod conjugate, the authors observed selective drug delivery to tumors while avoiding the surrounding, adjacent non-malignant tissue. Therapeutic application of the conjugate resulted in regression of the aggressive B16F10 melanoma lesions modeled in syngeneic mice.

In the study by Saghaeidehkordi et al., the authors targeted breast cancer cells with homing peptide 18-4 that targets cell surface Keratin 1 [8]. Administration of the peptide-doxorubicin conjugate in triple negative breast cancer mice resulted in improved delivery of the drug to the malignant lesions. Therapeutically, this improved delivery translated into significantly higher anti-tumor activity; mice treated with the conjugate showed improved antitumor efficacy and reduced side effects compared to control groups treated with doxorubicin or saline.

The reports in this Special Issue are important advances that illustrate the multifaceted and interdisciplinary efforts on development of peptide-based strategies for precision-guided therapy and diagnosis of disease. We wish to thank all the authors of this Special Issue for contributing their high-quality research and for the critical evaluation of their manuscripts.
